# Laparoscopic splenectomy in a case of blunt abdominal trauma

**DOI:** 10.4103/0972-9941.58503

**Published:** 2009

**Authors:** Arun Prasad, Narendra Agarwal

**Affiliations:** Department of Surgery, Apollo Hospital, Sarita Vihar, New Delhi, India

**Keywords:** Blunt abdominal trauma, laparoscopy India, splenectomy

## Abstract

Splenic rupture is a frequent consequence of blunt abdominal trauma. Removal of the spleen (splenectomy) or alternative conservative procedures (splenorrhaphy, partial splenectomy and haemostatic collagen application) are surgical treatment options. Splenectomy was first described in 1910 by Sutherland. Laparoscopic splenectomy was first described in 1991 by Delaitre and Maignien. Since then significant improvements in instrumentation and technology such as harmonic scalpel, endovascular staplers have increased its ease of performance. Laparoscopic splenectomy for a ruptured spleen has been reported only in a few cases, in which a hand-assisted technique was used. We present the first reported case from India (to the best of our knowledge) of a successful removal of a ruptured spleen by means of a totally laparoscopic technique.

## INTRODUCTION

Splenectomy was first described in 1910 by Sutherland.[1] Laparoscopic splenectomy was first described in 1991 by Delaitre and Maignien. Since then significant improvements in instrumentation and technology such as harmonic scalpel, endovascular staplers have increased its ease of performance.[2] Splenic rupture is a frequent consequence of blunt abdominal trauma. Removal of the spleen (splenectomy) or alternative conservative procedures (splenorrhaphy, partial splenectomy and haemostatic collagen application) are surgical treatment options.[3,4] Laparoscopic splenectomy for a ruptured spleen has been reported only in a few cases, in which a hand-assisted technique was used. We present the first reported case from India (to the best of our knowledge), of a successful removal of a ruptured spleen by means of a totally laparoscopic technique.

## CASE REPORT

**Figure 1:**
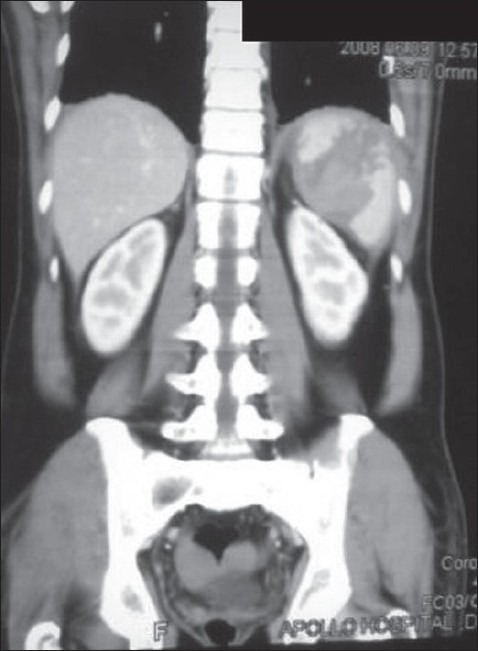
Pre-operative CT scan showing splenic injury and collection

A 46-year-old man was admitted to our hospital on an emergent basis after a motor vehicle trauma. His only complaint was of pain over the left flank. He did not have any peritoneal signs, and examination was unremarkable except for left flank tenderness. X-rays revealed fractures of left eighth and ninth ribs. Plain CT scan of thorax and abdomen confirmed the radiographic findings with evidence of splenic injury (Grade II and Grade III) with mild perisplenic collection [Figure 1]. On admission, he had an Hb of 14.1 g/dl. The patient was nursed in HDU and the CECT repeated on the next day revealed an increase in the size of splenic and perisplenic collection. The patient had a stable blood pressure but progressive tachycardia was noted. Subsequent blood pictures showed a decreasing trend of haemoglobin to 12.9 gm/dl and then to 11.8 gm/ dl. The various management options were discussed with the patient, including observation, angiographic embolization and splenectomy. We decided to proceed with diagnostic laparoscopy and splenectomy.

**Figure 2:**
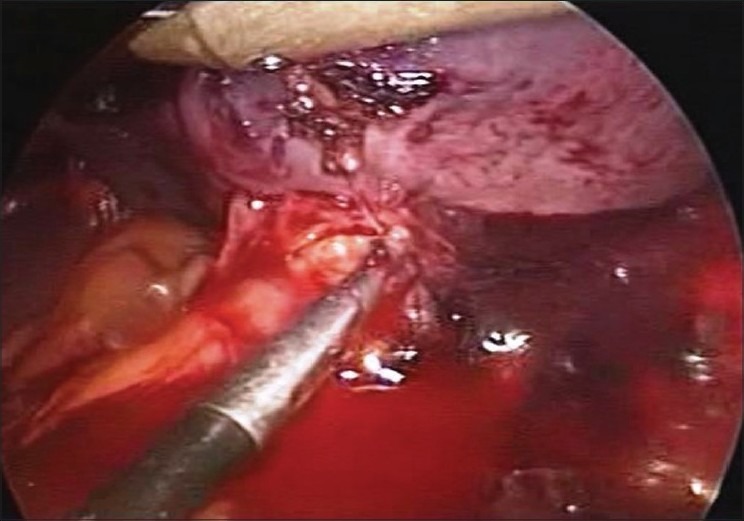
Peri-splenic collection at hilum with fresh ooze of blood

The patient was placed in the right lateral decubitus position. Two 10-mm ports and two 5-mm ports were used. A 10-mm camera and 5-mm atraumatic graspers were used for the initial dissection. A large haemoperitoneum was noted upon entering the abdomen. We were able to easily visualize the spleen, which was noted to have a large haematoma [Figure 2]. The Harmonic scalpel was then used to divide the ligamentous attachments of the spleen. The spleen was carefully retracted upward with a blunt grasper and the hilum was visualized. A 12-mm endoscopic stapler with a 60-mm blue cartridge was used to transect the hilum. No bleeding occurred from the remaining splenic artery or vein, and the tail of the pancreas was visualized and protected during this process. The superior short gastric arteries were also divided by using a harmonic scalpel. The spleen was removed by using a bag inserted through a 12-mm port. The spleen required morcellation to be removed due to its large size. The remaining haemoperitoneum was suctioned and a 32-Fr drain was placed in the splenic fossa. The patient was able to tolerate a regular diet on the second post-operative day with pain controlled by IV analgesics only. The drain was removed on the fifth post-operative day. The patient did not require any transfusions. He received his vaccinations for *Pneumococcus*, *Meningococcus* and *H*. *influenzae* during the hospital stay. He has remained well in follow-up visits.

## DISCUSSION

The management of blunt splenic injury has evolved considerably over the past decade. Mandatory laparotomy with splenorrhaphy or splenectomy has been replaced in most cases by nonoperative management. Failure rates for nonoperative treatment are very variable, ranging from 10% to 40%.[5] Patients with severe (Grade III and higher) splenic injuries have a higher failure rate than with injuries of lesser severity. They also have a higher transfusion requirement and increased morbidity and mortality in critically ill patients, including trauma victims.[6–8] Further risks of nonoperative treatment include delayed splenic rupture, persistent pain and prolonged immobilization which is contraindicated in a group at a high risk for venous thromboembolism. The need for bed rest and monitoring often requires prolonged hospital stays.

Splenic artery angio-embolization has been described as an alternative to operative management of splenic injuries. Lower grades of injury correspond to higher success rates for this approach.[9,10] Complications of angioembolization include the need for delayed splenectomy due to splenic infarction, infection, abscess or persistent pain.[11,12] The rate of these complications has been described as high as 33% in some series.[13]

Splenectomy is not indicated for all of the injuries, and angiographic embolization is an acceptable option to further define the injury and prophylactically reduce the rate of observation. The prevention of overwhelming postsplenectomy sepsis (OPSI) has often been cited as a reason for advocating splenic preservation. However, the incidence of OPSI in adults, in contrast with children, is very low (, 1%) and with modern vaccination and antibiotics is expected to decline further. In adult patients, the risks of transfusion and delayed splenic rupture, abscess and pain should be weighed against the risk of OPSI in determining the best approach to severe splenic injuries.

The role of laparoscopy in blunt trauma has yet to be defined. In experienced hands, it has been shown to reduce the negative laparotomy rate and identify and treat diaphragmatic and visceral injuries.[14] Several authors have used laparoscopy to apply haemostatic agents to solid organ lacerations and perform spleen-preserving procedures in lower grade injury.[15] We believe that if there is a splenic injury and no other injury in the abdomen, as shown by CT and FAST scans, the laparoscopic approach is feasible, even though the gold standard is still considered exploratory laparotomy. Laparoscopic splenectomy has not been well described in patients with splenic trauma. Nasr *et al*.[3] reported the successful use of laparoscopic splenectomy in four patients. These patients were all stable with isolated splenic injuries. They were operated on after an initial period of stabilization and nonoperative treatment. Two patients had a Grade III and one had a Grade I injury. One patient had previously undergone splenic artery embolization and re-presented with delayed splenic rupture. The grade of injury in this case was not stated. They had no complications from the laparoscopic approach. Based on our experience, we would like to point out that laparoscopic splenectomy can be safely performed in a high-grade injury by an experienced surgeon and should be considered in stable patients. A single injury to the spleen with no other injuries reported by CT and FAST scans in patients who are haemodynamically stable could be an indication for laparoscopic splenectomy. In our case, Grade II and Grade III injuries were reported with the CT scan, and there was a big haematoma at the level of the hilum. Though there is nothing in the literature that suggests immediate splenectomy in a young, healthy, stable patient with a high-grade injury based on the CT scan, but laparoscopic exploration in trauma[11] is an acceptable option. Patients with high-grade splenic injuries are more likely to fail conservative treatment. Nonoperative treatment is associated with several risks, including the risk of blood transfusion. Transfusion is being increasingly recognized as an independent factor in poor outcomes, and avoidance of transfusion is likely to become a priority in managing stable trauma patients. Laparoscopic splenectomy should be considered as an option in these patients, either on admission or after failure of nonoperative management.

Partial laparoscopic splenectomy has been reported in patients with localized benign or malignant disease of the spleen but its role in blunt trauma still needs to be delineated.

We believe that with the laparoscopic technique, the experience of the surgeon is crucial; however, there is no consensus to who should attempt laparoscopic splenectomy and who should not.

An availability of open surgery instruments is necessary and if needed there should be no hesitation in entering the abdomen by a left subcostal incision that can be easily done in the right lateral position that we use for laparoscopic splenectomy.

Our patient remained in the hospital for 6 days solely for observation. He was able to resume a regular diet on the second day and had minimal requirements for pain medication.

## CONCLUSION

Laparoscopic splenectomy can be successfully used as an immediate treatment option in haemodynamically stable patients with severe splenic injury.
